# Coherently excited superresolution using intensity product of phase-controlled quantum erasers via polarization-basis projection measurements

**DOI:** 10.1038/s41598-024-62144-6

**Published:** 2024-05-21

**Authors:** Byoung S. Ham

**Affiliations:** 1 School of Electrical Engineering and Computer Science, Gwangju Institute of Science and Technology, 123 Chumdangwagi-ro, Buk-gu, Gwangju, 61005 South Korea; 2 Qu-Lidar, 123 Chumdangwagi-ro, Buk-gu, Gwangju, 61005 South Korea

**Keywords:** Quantum metrology, Optical physics

## Abstract

Recently, the delayed-choice quantum eraser has been applied for coherently excited superresolution using phase-controlled projection measurements of laser light to overcome the diffraction limit in classical physics as well as to solve the limited order N of the N00N state in quantum physics. Here, a general scheme of the phase-controlled quantum eraser-based superresolution is proposed for quantum sensing satisfying the Heisenberg limit, and its general solution is derived for an arbitrary Nth-order intensity correlation. Furthermore, phase quantization of the proposed superresolution is discussed to better understand the wave nature of quantum mechanics. Unlike other methods of superresolution in quantum sensing, the proposed method is for the intensity products between phase-controlled quantum erasers and thus is compatible with most conventional sensing metrologies.

## Introduction

Quantum entanglement is between two or more individual particles, where a fixed phase relation between paired photons does not violate quantum mechanics^1^. A typical method of entangled photon pair generation is to use a spontaneous parametric down-conversion process^2^, where the phase-matching condition among the pump and two sibling photons is critical^1–4^. Unlike a single photon, thus, the fixed phase between entangled photons is straightforward for the wave nature of quantum mechanics^1–7^. Such an understanding of the wave nature-based quantum correlation has emerged to revisit the Hong-Ou-Mandel (HOM) effect^5,6^, Franson-type nonlocal correlation^7^, and delayed-choice quantum eraser^8,9^. Experimental demonstration of the fixed phase relation has been conducted in trapped ions for a $\uppi /2$ phase difference^10^. A complete coherence solution of the HOM effect for the $\uppi /2$ phase relation has also been presented^5,6^. Most recently, the same phase relation has been applied to superresolution in quantum sensing whose fundamental physics is in the nonlocal correlation^11^.

The wave-particle duality originates in quantum superposition, where these two natures are mutually exclusive^12–14^. In a single photon’s self-interference^15^, thus, the quantum superposition is between orthonormal bases of the single photon^16–21^. With the wave nature, the delayed-choice quantum eraser^17^ has been newly interpreted^22^, as an ad-hoc quantum superposition of orthonormal bases of a single photon through a dynamic window of a polarizer for the basis-projection measurement^23^. Due to the exclusive nature between the phase (wave) and photon number (particle), thus, the interpretation of the quantum eraser represents a deterministic quantum feature, where no difference exists between single photon^8^ and continuous wave (cw) light due to the first-order intensity correlation^9,24^. Similarly, phase-controlled superresolution^25^ has been experimentally demonstrated using a single photon and cw light for the same quantum feature of photonic de Broglie waves (PBWs)^26–32^.

Here, a universal scheme of the phase-controlled superresolution is proposed for quantum sensing using a cw laser in a Michelson interferometer. In quantum sensing and metrology, the superresolution overcoming the shot-noise limit (SNL) has been experimentally demonstrated using higher-order entangled photons, i.e., N00N states to satisfy the Heisenberg limit (HL)^26–32^. The N00N state-based superresolution is known as PBWs^26–32^. Unlike N00N states, however, squeezed states cannot be used for superresolution but supersensitivity^34^. The PBW-like superresolution effect has also been observed using phase-controlled coherent photons in a noninterferometric system^35,36^ via projection measurements^23^. On the contrary, the proposed superresolution is for the intensity product of phase-controlled quantum erasers using a quarter-wave plate (QWP) in a classical (coherence) regime of light^11,25^. In this article, a universal scheme of an arbitrary Nth-order superresolution is proposed, and its general solution is coherently derived for the intensity product of quantum erasers via projection measurements. Finally, the superresolution is compared with PBW-like quantum features and discussed for phase quantization of the ordered intensity products in the viewpoint of the wave nature of quantum mechanics.

## Result

### Phase-controlled projection measurement of quantum erasers for superresolution

**Figure 1:**
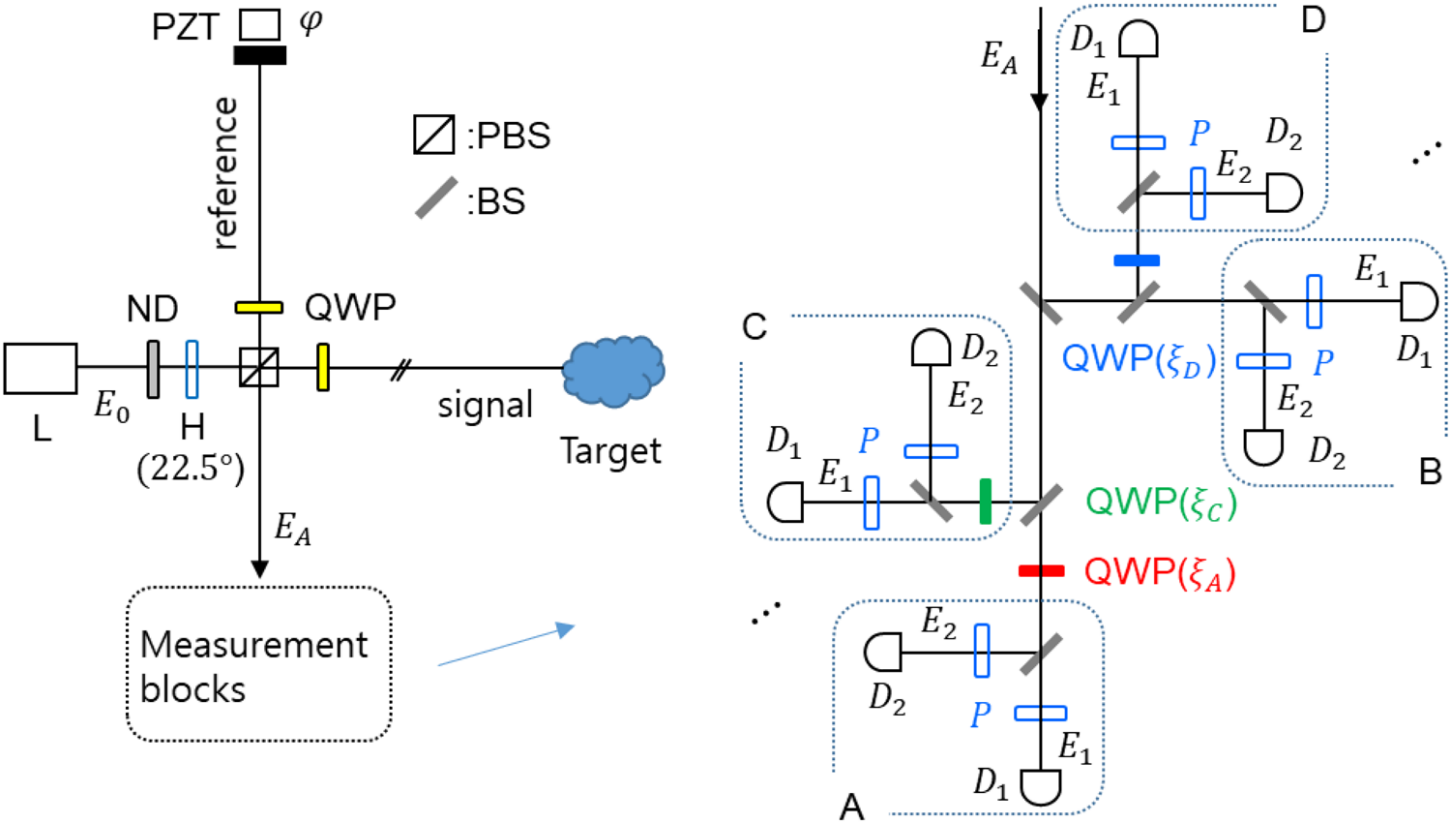
Schematic of a universal super-resolution based on phase-controlled quantum erasers. L: laser, ND: neutral density filter, H: half-wave plate, PBS: polarizing beam splitter, PZT: piezo-electric transducer, QWP: quarter-wave plate, P: polarizer, D: single photon (or photo-) detector, All rotation angles of Ps are at $\uptheta =45^\circ$.

**Figure 2:**
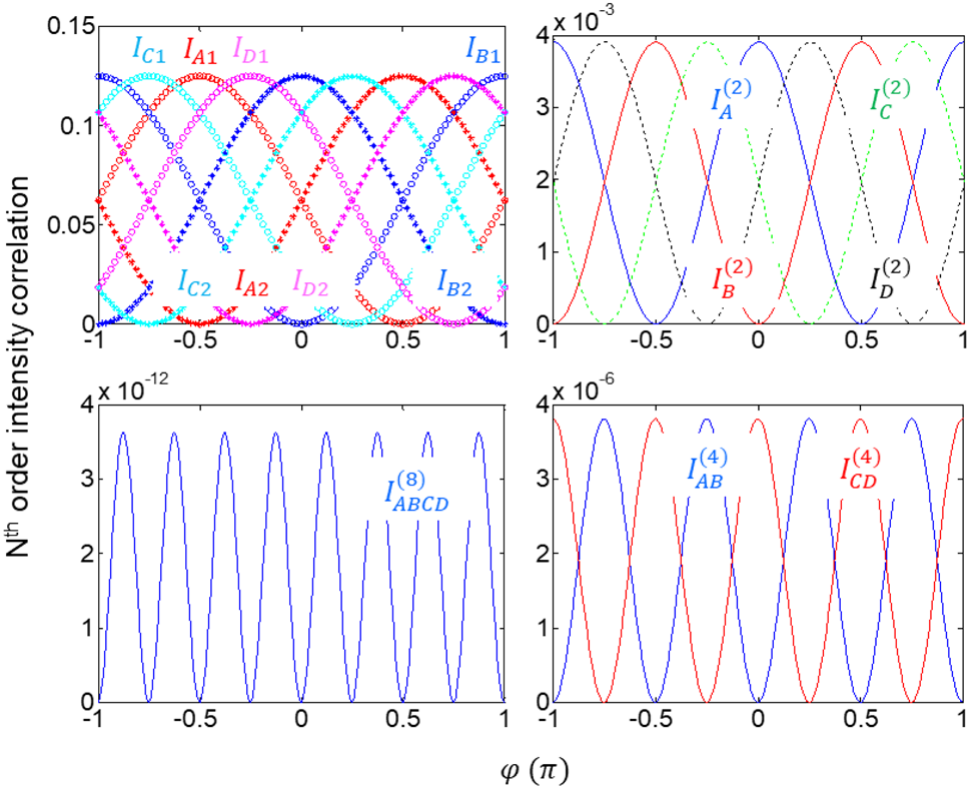
Numerical calculations of the Nth order intensity correlations in Fig. 1. (upper left) Individual first-order intensity correlation ${I}_{j}$ in A, B, C, and D blocks. Blue star (circle): B_3_ (B_4_) in B, Cyan star (circle): C_3_ (C_4_) in C, Red star (circle): A_3_ (A_4_) in A, Magenta star (circle): D_3_ (D_4_) in D. (upper right) Second-order intensity correlation in each block of the Inset of Fig. 1. (lower right) Fourth-order intensity correlation between (red) A and B, and (blue) C and D. (lower left) Eight-order intensity product between all quantum erasers. ${I}_{K}={I}_{K1}{I}_{K2}$ (K = A, B, C, D), ${I}_{AB}^{(4)}={I}_{A}^{(2)}{I}_{B}^{(2)}$, ${I}_{CD}^{(4)}={I}_{C}^{(2)}{I}_{D}^{(2)}$, and ${I}_{ABCD}^{(8)}={I}_{AB}^{(4)}{I}_{CD}^{(4)}$. ${\xi }_{A}=\frac{\pi }{2}$, ${\xi }_{C}=\frac{\pi }{4}$, and ${\xi }_{D}=\frac{3\pi }{4}$.

Figure 1 shows a universal scheme of the classically (coherently) excited superresolution based on phase-controlled quantum erasers. The superresolution scheme in Fig. 1 originates in the Nth-order intensity correlations between phase-controlled quantum erasers, resulting in the PBW-like quantum feature^11,25^, as shown in Fig. 2. Compared to the N = 4 case^11,25^, the Inset of Fig. 1 shows an arbitrary Nth-order superresolution scheme, where the first eight quantum erasers for N = 8 are visualized with dotted blocks to explain the cascaded phase control of the quantum erasers using QWPs. For the quantum eraser, both single photon^8^ and cw laser light^9^ were experimentally demonstrated in a Mach–Zehnder interferometer (MZI) for the polarization-basis projection onto a polarizer P. The MZI physics of coherence optics^37^ shows the same feature in both a single photon^15^ and cw light due to the limited Sorkin parameter, as discussed for the Born rule tests^38^. This originates in the equality between quantum and classical approaches for the first-order (N = 1) intensity correlation^24^. Quantum mechanically, the deterministic feature of the MZI system is due to the double unitary transformation of a 50/50 nonpolarizing beam splitter (BS)^1,15^. The use of neutral density filters is not to generate single photons but to protect photodiodes from intensity saturation.

The rotation angle of QWP in each block of the quantum erasers in the Inset of Fig. 1 is to induce a phase gains (${\xi }_{j})$ to the vertical component of the corresponding light^37^. As experimentally demonstrated^25^, the QWP induces a phase delay to the vertical polarization component compared to the horizontal one^37^. This polarization-basis-dependent phase gain of the light directly affects the quantum eraser via polarization-basis projection measurements, resulting in a fringe shift^11,25^, because the role of the polarizer P is to project orthogonal polarization bases onto the common axis $\widehat{{\text{p}}}$ (see Eqs. (2)–(8))^8,9,18^. The random path length to the polarizer from PBS in Fig. 1 does not influence the intensity correlations due to the unaffected global phase by the Born rule, where intensity (measurement) is the absolute square of the amplitude^13,14^. Thus, controlling the QWP of each block makes an appropriate fringe shift of the quantum erasers for the first-order intensity products.

**Figure 3:**
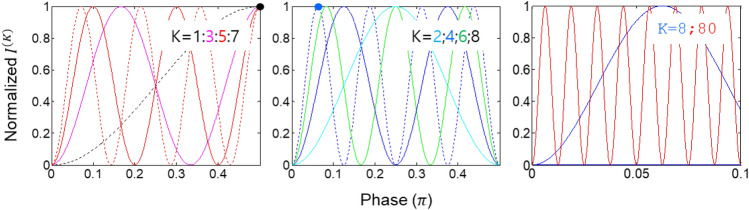
Numerical calculations for the normalized Kth-order intensity products. K represents the number of quantum erasers used for intensity product measurements.

**Figure 4:**
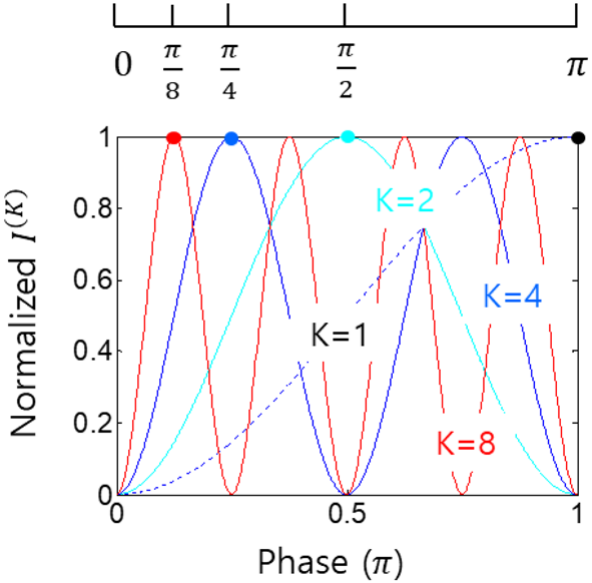
Phase quantization of the intensity products in Fig. 3. K is the order of intensity product. Dotted: K = 1, Cyan: K = 2, Blue: K = 4, Red: K = 8.

In the proposed universal scheme with a practically infinite number of phase-controlled quantum erasers in Fig. 1, a general coherence solution of the phase-controlled superresolution is coherently derived from the combinations of QWPs (see Eq. (25) and Figs. 2 and 3). Then, the general solution is compared with PBWs based on N00N states for the discussion of phase quantization of the Nth-order intensity product in Fig. 4. Such phase quantization has already been separately discussed for coherence de Broglie waves (CBWs) in a coupled MZI system for the wave nature of quantum mechanics^39,40^. Unlike CBWs resulting from MZI superposition, the present phase quantization of superresolution is for the intensity product between phase-controlled quantum erasers. On the contrary to energy quantization of the particle nature in quantum mechanics^1^, the phase quantization is for the wave nature, where the particle and wave natures are mutually exclusive.

### Analysis 1: PBW-like superresolution

A coherence approach based on the wave nature of a photon is adopted to analyze Fig. 1 differently from the quantum approach based on quantum operators^1,26–33^. The novel feature of the present method is to use common intensity products of cw lights via polarization-basis projection of the phase-controlled quantum erasers. Thus, there is no need for single-photon coincidence detection. Instead, the intensity product is enough for a single shot measurement, as is in nonlinear optics. Technically, the condition ${\text{N}}\le {\text{M}}$ is required, where N and M are the number of quantum erasers used for the intensity product and the photon number of the input light, respectively. Here it should be noted that both intensity product and coincidence detection are effective within the ensemble coherence time of the input light L. In that sense, a pulsed laser is more appropriate for the use of a time-bin scheme as shown for quantum key distribution^41^.

The amplitude of the output field of the Michelson interferometer in Fig. 1 is represented using the BS matrix representation^42^ as:

1 $${{\varvec{E}}}_{A}=\frac{i{E}_{0}}{\sqrt{2}}\left(\widehat{H}{e}^{i\varphi }+\widehat{V}\right)$$

where ${E}_{0}$ is the amplitude of the light just before entering the Michelson interferometer. $\widehat{H}$ and $\widehat{V}$ are unit vectors of horizontal and vertical polarization bases of the light, respectively. In Eq. (1), the original polarization bases are swapped by the 45° rotated QWPs inserted in both paths for full throughput to the ${E}_{A}$ direction. Due to the orthogonal bases, Eq. (1) results in no fringe, satisfying the distinguishable photon characteristics of the particle nature in quantum mechanics: $\langle {I}_{A}\rangle ={I}_{0}$.

By the rotated polarizers in Fig. 1, whose rotation angle $\uptheta$ is from the horizontal axis, Eq. (1) is modified for the split quantum erasers:

2 $${{\varvec{E}}}_{A1}=\frac{i{E}_{0}}{\sqrt{2}\sqrt{8}}\left(cos\theta {e}^{i\varphi }+sin\theta {e}^{i{\xi }_{A}}\right)\widehat{p}$$

3 $${{\varvec{E}}}_{A2}=\frac{-{E}_{0}}{\sqrt{2}\sqrt{8}}\left(-cos\theta {e}^{i\varphi }+sin\theta {e}^{i{\xi }_{A}}\right)\widehat{p}$$

4 $${{\varvec{E}}}_{B1}=\frac{-i{E}_{0}}{\sqrt{2}\sqrt{8}}\left(cos\theta {e}^{i\varphi }+sin\theta \right)\widehat{p}$$

5 $${{\varvec{E}}}_{B2}=\frac{-i{E}_{0}}{\sqrt{2}\sqrt{8}}\left(-cos\theta {e}^{i\varphi }+sin\theta \right)\widehat{p}$$

6 $${{\varvec{E}}}_{C1}=\frac{-{E}_{0}}{\sqrt{2}\sqrt{8}}\left(cos\theta {e}^{i\varphi }+sin\theta {e}^{i{\xi }_{C}}\right)\widehat{p}$$

7 $${{\varvec{E}}}_{C2}=\frac{-i{E}_{0}}{\sqrt{2}\sqrt{8}}\left(-cos\theta {e}^{i\varphi }+sin\theta {e}^{i{\xi }_{C}}\right)\widehat{p}$$

8 $${{\varvec{E}}}_{D1}=\frac{-i{E}_{0}}{\sqrt{2}\sqrt{8}}\left(cos\theta {e}^{i\varphi }+sin\theta {e}^{i{\xi }_{D}}\right)\widehat{p}$$

9 $${{\varvec{E}}}_{D2}=\frac{{E}_{0}}{\sqrt{2}\sqrt{8}}\left(-cos\theta {e}^{i\varphi }+sin\theta {e}^{i{\xi }_{D}}\right)\widehat{p}$$

where $\widehat{p}$ is the axis of the polarizers, and $\sqrt{8}$ is due to the eight divisions (N = 8) of ${{\varvec{E}}}_{A}$ by the lossless BSs. In Eqs. (2)–(9), the projection onto the polarizer results in $\widehat{H}\to cos\theta \widehat{p}$ and $\widehat{V}\to sin\theta \widehat{p}$. By BS, the polarization direction of $\widehat{H}$ is reversed, as shown in the mirror image^37^. By the inserted QWP in each block, the ${\xi }_{j}$-dependent phase gain is to the $\widehat{V}$ component only^37^. As demonstrated for the projection measurement of N interfering entangled photons^23,29^, the Nth-order intensity correlation is conducted by the N split ports in the Inset of Fig. 1.

Thus, the corresponding mean intensities of all QWP-controlled quantum erasers in the Inset of Fig. 1 are as follows for $\uptheta =45^\circ$ of all Ps:

10 $$\langle {I}_{A1}\rangle =\frac{{I}_{0}}{2N}\langle 1+{{\cos}}(\varphi -{\xi }_{A})\rangle$$

11 $$\langle {I}_{A2}\rangle =\frac{{I}_{0}}{2N}\langle 1-{{\cos}}(\varphi -{\xi }_{A})\rangle$$

12 $$\langle {I}_{B1}\rangle =\frac{{I}_{0}}{2N}\langle 1+cos\varphi \rangle$$

13 $$\langle {I}_{B2}\rangle =\frac{{I}_{0}}{2N}\langle 1-cos\varphi \rangle$$

14 $$\langle {I}_{C1}\rangle =\frac{{I}_{0}}{2N}\left\langle 1+{\cos}(\varphi -{\xi }_{C})\right\rangle$$

15 $$\langle {I}_{C2}\rangle =\frac{{I}_{0}}{2N}\left\langle 1-{\cos}(\varphi -{\xi }_{C})\right\rangle$$

16 $$\langle {I}_{D1}\rangle =\frac{{I}_{0}}{2N}\left\langle 1+{\cos}(\varphi -{\xi }_{D})\right\rangle$$

17 $$\langle {I}_{D2}\rangle =\frac{{I}_{0}}{2N}\left\langle 1-{\cos}(\varphi -{\xi }_{D})\right\rangle$$

Equations (10)–(17) are the unveiled quantum mystery of the cause-effect relation of the quantum eraser found in the ad-hoc polarization-basis superposition via the polarization projection onto the $\widehat{p}$ axis of the polarizer. The price to pay for this quantum mystery is 50% photon loss by the polarization projection^11,22^, regardless of single photons^8^ or cw light^9^. By adjusting ${\xi }_{j}$ of QWP in each block, appropriate fringe shifts of the quantum erasers can also be made accordingly, as shown in Fig. 2 for ${\xi }_{A}=\frac{\pi }{2}$, ${\xi }_{C}=\frac{\pi }{4}$, and ${\xi }_{D}=\frac{3\pi }{4}$.

The corresponding second-order (N = 2) intensity correlations between the quantum erasers in each block is directly obtained from Eqs. (10)–(17) for ${\xi }_{A}=\frac{\pi }{2}$, ${\xi }_{C}=\frac{\pi }{4}$, and ${\xi }_{D}=\frac{3\pi }{4}$:

18 $$\left\langle {{\text{I}}}_{A1A2}^{(2)}(0)\right\rangle ={\left(\frac{{I}_{0}}{2N}\right)}^{2}\left\langle {sin}^{2}\left(\varphi -\frac{\pi }{2}\right)\right\rangle$$

19 $$\left\langle {{\text{I}}}_{B1B2}^{(2)}(0)\right\rangle ={\left(\frac{{I}_{0}}{2N}\right)}^{2}\left\langle {sin}^{2}\varphi \right\rangle$$

20 $$\left\langle {{\text{I}}}_{C1C2}^{(2)}(0)\right\rangle ={\left(\frac{{I}_{0}}{2N}\right)}^{2}\left\langle {sin}^{2}\left(\varphi -\frac{\pi }{4}\right)\right\rangle$$

21 $$\left\langle {{\text{I}}}_{D1D2}^{(2)}(0)\right\rangle ={\left(\frac{{I}_{0}}{2N}\right)}^{2}\left\langle {sin}^{2}\left(\varphi -\frac{3\pi }{4}\right)\right\rangle$$

where the second-order intensity fringes are also equally shifted as in the first-order fringes (see Fig. 2). Likewise, the fourth-order (N = 4) intensity correlations between any two blocks can be derived from Eqs. (18)–(21) as:

22 $$\left\langle {{\text{I}}}_{A1A2B1B2}^{(4)}(0)\right\rangle ={\left(\frac{{I}_{0}}{2N}\right)}^{4}\left\langle {sin}^{2}\varphi {sin}^{2}\left(\varphi -\frac{\pi }{2}\right)\right\rangle$$

23 $$\left\langle {{\text{I}}}_{C1C2D1D2}^{(4)}(0)\right\rangle ={\left(\frac{{I}_{0}}{2N}\right)}^{4}\left\langle {sin}^{2}\left(\varphi -\frac{\pi }{4}\right){sin}^{2}\left(\varphi -\frac{3\pi }{4}\right)\right\rangle$$

Thus, the eighth-order (N = 8) intensity correlation for all quantum erasers in the Inset of Fig. 1 is represented as:

24 $$\left\langle {{\text{I}}}_{A1A2B1B2C1C2D1D2}^{(8)}(0)\right\rangle ={\left(\frac{{I}_{0}}{2N}\right)}^{8}\left\langle {sin}^{2}\varphi {sin}^{2}\left(\varphi -\frac{\pi }{4}\right){sin}^{2}\left(\varphi -\frac{\pi }{2}\right){sin}^{2}\left(\varphi -\frac{3\pi }{4}\right)\right\rangle$$

From Eq. (24), the proposed scheme of superresolution for N = 8 is analytically confirmed for the satisfaction of the Heisenberg limit in quantum sensing (see Figs. 2 and 3).

### Analysis 2: Numerical calculations of the superresolution

Figure 2 shows numerical calculations of the Nth-order intensity correlations using Eqs. (10)–(17) for ${\xi }_{A}=\uppi /2$, ${\xi }_{C}=\uppi /4$, and ${\xi }_{D}=3\uppi /4$ to demonstrate the proposed PBW-like superresolution using phase-controlled coherent light in Fig. 1. From the upper-left panel to the clockwise direction in Fig. 2, the simulation results are shown for ordered (N = 1, 2, 4, 8) intensity correlations. As shown, all ordered-intensity correlations are equally spaced in the phase domain, where the pair of quantum erasers in each block satisfies the out-of-phase relation (see the same colored ‘o’ and ‘*’ curves in the upper-left panel). Thus, the higher-order intensity correlation between blocks also results in the same out-of-phase relation, as shown for N = 2 and N = 4, resulting in the Heisenberg limit, $\mathrm{\delta \varphi }=\uppi /{\text{N}}$.

For an arbitrary order N, the jth block with ${\xi }_{j}$-QWP can be assigned to the universal scheme of the phase-controlled superresolution. For the expandable finite block series with ${\xi }_{j}$-phase-controlled quantum erasers in Fig. 1, the generalized solution of the kth-order intensity correlation can be quickly deduced from Eq. (24):

25 $$\left\langle {{\text{I}}}^{(K)}(0)\right\rangle ={\left(\frac{{I}_{0}}{2N}\right)}^{K}\left\langle \prod_{j=0}^{K}{sin}^{2}(\varphi -{\xi }_{j})\right\rangle$$

where ${\xi }_{j}=j2\pi /N$ and ${\text{K}}\le {\text{N}}$. Unlike the N00N-based superresolution in quantum sensing^26–31^, the kth-order intensity product in Eq. (25) can be coherently amplified as usual in classical (coherence) sensors. Thus, the reduction by ${\left(\frac{{I}_{0}}{2N}\right)}^{k}$ has no critical problem for potential applications of the proposed superresolution.

Figure 3 is for the details of numerical calculations for K = 1,2,...,8 and K = 80 using Eq. (25). The top panels of Fig. 3 are for odd and even Ks, where the fringe number linearly increases as K increases, satisfying the Heisenberg limit^31^. For the K-proportional fringe numbers, the positions of the first fringes for K = 1,2,...,8 move from $\uppi /2$ for K = 1 (black dot, left panel) to $\uppi /16$ for K = 8 (blue dot, middle panel). As in PBWs, thus, the same interpretation of the K-times increased effective frequency to the original frequency of the input light can be made for the Kth-order intensity correlations. Unlike N00N state-based PBWs, the intensity-product order can be post-determined by choosing K detectors out of N quantum erasers.

The right panel of Fig. 3 is for comparison purposes between K = 8 and K = 80, where the resulting ten times increased fringe numbers indicate ten times enhanced phase resolution, satisfying the Heisenberg limit. Thus, the pure coherence solution of the PBW-like quantum feature satisfying the Heisenberg limit is numerically confirmed for the generalized solution of Eq. (25). Here, the coincidence detection in the particle nature of quantum sensing with N00N states is equivalent to the coherence intensity-product measurement, where the coherence between quantum erasers is provided by the cw laser L within its spectral bandwidth. Furthermore, the ${\xi }_{j}$ relation between blocks composed of paired quantum erasers may imply the phase relation between paired entangled photons (discussed elsewhere).

Figure 4 discusses the perspective of the phase-basis relation provided by ${\xi }_{j}$ in Eq. (25) for the Kth-order intensity correlations of the proposed superresolution. From the colored dots representing the first fringes of the ordered intensity products, the generalized phase basis of the Kth-order intensity correlation can be deduced for ${\mathrm{\varphi }}_{K}=\uppi /{\text{K}}$. Thus, the Kth-order intensity correlation behaves as a K-times increased frequency ${f}_{K} (=K{f}_{0})$ to the original input frequency ${f}_{0}$ of L. The intensity-order dependent effective frequency ${f}_{K}$ is equivalent to the PBW of the N00N state in quantum metrology^26–32^.

Based on the K-times increased fringes in the Kth-order intensity product, the numerical simulations conducted in Fig. 4 can be interpreted as phase quantization of the intensity products through projection measurements of the quantum erasers. As shown in the PBW-like quantum features, these discrete eigenbases of the intensity products can also be compared to a K-coupled pendulum system^43^, where the phase quantization in Fig. 4 can be classically understood^39,40^. Unlike the N-coupled pendulum system^43^ or CBWs from MZI interference^39,40^, however, any specific mode of ${\varphi }_{K}$ can be deterministically taken out by post-selection of a particular number of blocks used for the intensity-product order K in Fig. 1. Like the energy quantization of the particle nature in quantum mechanics, thus, Fig. 4 is another viewpoint of the wave nature for the proposed superresolution. By the wave-particle duality in quantum mechanics, both features of the energy and phase quantization are mutually exclusive.

From the universal scheme of the superresolution based on the phase-controlled quantum erasers in Fig. 1, a generalized solution of the Kth-order intensity correlation in Fig. 4 can also be intuitively obtained:

26 $$\left\langle {{\text{I}}}_{{P}_{1}{P}_{2}\dots {P}_{j}\dots {P}_{K/2}}^{(K)}(0)\right\rangle ={\left(\frac{{I}_{0}}{2N}\right)}^{K}\left\langle {sin}^{2}(K\varphi /2)\right\rangle$$

where ${P}_{j}={Z}_{1}{Z}_{2}$, and ${Z}_{j}$ is the jth quantum eraser of the P block. Here, the effective phase term $K\varphi$ in Eq. (26) represents the typical nonclassical feature of PBWs used for quantum sensing with N00N states ^30,31^. The numerical simulations of Eq. (26) for N = 1, 2, 4, and 8 perfectly match those in Fig. 4 (not shown). Although the mathematical forms between Eqs. (25) and (26) are completely different, their quantum behaviors are the same as each other. Thus, Eq. (26) is equivalent to the superresolution in Eq. (25) ^13,25^, where the phase quantization is accomplished by ordered intensity products of the divided output fields of the Michelson interferometer. Unlike coincidence detection between entangled photons under the particle nature^26–32^, the present coherence scheme with the wave nature is intrinsically deterministic within the spectral bandwidth of the input laser. Thus, the coincidence detection in N00N-based quantum sensing is now replaced by the intensity product between independently phase-controlled quantum erasers using QWPs. Such a coherence technique of the individually and independently controlled quantum erasers can be applied for a time-bin scheme with a pulsed laser, where intensity products between different time bins are completely ignored due to their incoherence feature^41^.

## Conclusion

A universal scheme of superresolution was presented for the intensity product of the phase-controlled quantum erasers via polarization-basis projection measurements in a Michelson interferometer. The related general solution of the superresolution was also coherently derived from the universal scheme of the intensity product between phase-controlled quantum erasers. For the phase control of the quantum erasers, QWPs were assigned to N-divided output fields of the interferometer by discretely setting their rotation angles. As a result, all individual first-order intensity correlations of the N phase-controlled quantum erasers satisfied non-overlapped and equally spaced fringes in the phase domain, resulting in superresolution of the PBW-like quantum feature for their higher-order intensity products. Furthermore, the ordered intensity products were interpreted as phase quantization in a viewpoint of the wave nature in quantum mechanics, as the energy quantization corresponds to the particle nature. Thus, the phase-controlled superresolution was inherently deterministic due to the wave nature. Although such quantized phase modes were found in an N-coupled pendulum system, a deterministic choice of a particular eigenmode of the superresolution was possible in the proposed superresolution by post-selection of a particular number of quantum erasers. Thus, the coherence quantum feature of the proposed superresolution may open the door to new quantum sensing and metrology to overcome the limited N00N state-based quantum sensing. The intuitively deduced phase quantization of the ordered intensity products for the proposed superresolution should intrigue a new quantum technology compatible with coherence optics.

## Methods

The polarizing beam splitter (PBS) of the Michelson interferometer in Fig. 1 provides random polarization bases of the input photon (light) via a 22.5° rotated half-wave plate, where the role of the polarizers in the measurement blocks (see the Inset) is for the projection measurement of the polarization bases. By PBS, polarization bases of the light are correlated to the paths of the Michelson interferometer. The vertical-basis of the input photon (light) is used as ‘reference,’ while the horizontal-basis is used as ‘signal’ to detect an unknown object ‘target.’ For the full collection of both reference and signal photons into one output path (${E}_{A}$), a quarter-wave plate is inserted in each path. For the intensity product, the output path is divided into N sub-paths, where each path corresponds to each quantum eraser. For the phase control of the quantum eraser set in each block (see the dotted box in the Inset), a quarter-wave plate (QWP) is inserted, where the reference is set for block B without QWP. For equal fringe spacing among the quantum erasers, the rotation angles of QWPs are appropriately adjusted, as shown by the phase quantization in Fig. 4. The maximum number N of the port division is ideally up to the photon number of the input light, where 10^15^ photons are for a 1 mW HeNe laser. For the intensity product between quantum erasers, a pulsed laser scheme may be applied to distinguish different time-bin pulses, where the physical distance of the (signal) path in the Michelson interferometer can be easily set to be beyond the light corn determined by the measurement time of a photodiode. This condition satisfies the violation of the cause-effect relation in the quantum eraser.

## Data Availability

All data generated or analyzed during this study are included in this published article.
